# Treatment of haemorrhoids: rubber band ligation or sclerotherapy (THROS)? Study protocol for a multicentre, non-inferiority, randomised controlled trial

**DOI:** 10.1186/s13063-023-07400-2

**Published:** 2023-06-03

**Authors:** J. Y. van Oostendorp, T. C. Sluckin, I. J. M. Han-Geurts, S. van Dieren, R. Schouten, on behalf of the THROS trial research group

**Affiliations:** 1 Department of Surgery, Amsterdam University Medical Centers, Location AMC, Meibergdreef 9, 1105 AZ Amsterdam, the Netherlands; 2 Proctos Kliniek, Prof. Bronkhorstlaan 10, 3823 MB Bilthoven, The Netherlands; 3 Department of Surgery, Flevoziekenhuis, Hospitaalweg 1, 1315 RA Almere, The Netherlands

**Keywords:** Haemorrhoidal disease, Grade 1–2 haemorrhoids, Rubber band ligation, Sclerotherapy, Patient-related outcome measures, PROMs

## Abstract

**Introduction:**

Haemorrhoidal disease (HD) is a common condition with significant epidemiologic and economic implications. While it is possible to treat symptomatic grade 1–2 haemorrhoids with rubber band ligation (RBL) or sclerotherapy (SCL), the effectiveness of these treatments compatible with current standards has not yet been investigated with a randomised controlled trial. The hypothesis is that SCL is not inferior to RBL in terms of symptom reduction (patient-related outcome measures (PROMs)), patient experience, complications or recurrence rate.

**Methods and analysis:**

This protocol describes the methodology of a non-inferiority, multicentre, randomised controlled trial comparing rubber band ligation and sclerotherapy for symptomatic grade 1–2 haemorrhoids in adults (> 18 years). Patients are preferably randomised between the two treatment arms. However, patients with a strong preference for one of the treatments and refuse randomisation are eligible for the registration arm. Patients either receive 4 cc Aethoxysklerol 3% SCL or 3 × RBL. The primary outcome measures are symptom reduction by means of PROMs, recurrence and complication rates. Secondary outcome measures are patient experience, number of treatments and days of sick leave from work. Data are collected at 4 different time points.

**Discussion:**

The THROS trial is the first large multicentre randomised trial to study the difference in effectivity between RBL and SCL for the treatment of grade 1–2 HD. It will provide information as to which treatment method (RBL or SCL) is the most effective, gives fewer complications and is experienced by the patient as the best option.

**Ethics and dissemination:**

The study protocol has been approved by the Medical Ethics Review Committee of the Amsterdam University Medical Centers, location AMC (nr. 2020_053). The gathered data and results will be submitted for publication in peer-reviewed journals and spread to coloproctological associations and guidelines.

**Trial registration:**

Dutch Trial Register NL8377. Registered on 12–02-2020.

**Supplementary Information:**

The online version contains supplementary material available at 10.1186/s13063-023-07400-2.

## Significance statement

Haemorrhoidal disease (HD) is a common condition with significant epidemiologic and economic relevance. Until now, there are no large multicentre randomised trials that have studied the difference in effectivity and patient experience between RBL and SCL for the treatment of grade 1–2 HD.

## Introduction

### Background and rationale

Haemorrhoids are a part of normal anatomy, and yet the term haemorrhoids is often used to refer to haemorrhoidal disease (HD) in which clusters of vascular tissue, smooth muscle and connective tissue in the anal canal give rise to symptoms of blood loss, itchiness, soiling and/or prolapse [1]. HD is a common condition with significant epidemiologic and economic relevance. The prevalence of HD rises up to 55% for adults aged between 45 and 65 years of age, with an annual incidence of 5% of the general population [2].

In 1985, Goligher was the first to report a widely accepted grading system for haemorrhoids, which describes four separate groups based on the extent of prolapse inside, or outside, the anal canal [3]. In 460 B.C., Hippocrates described HD with a plethora of treatments, varying from burning, herbs, ligation and surgical excision [4]. Currently, various treatments are available to treat HD [5, 6].

Symptomatic grade 1–2 haemorrhoids can be treated with either rubber band ligation (RBL) or sclerotherapy (SCL) and both treatment options often require multiple sessions [6]. The most common symptom for this patient population is rectal blood loss accompanying defecation [3, 7]. RBL is based on the strangulation and necrosis of haemorrhoidal tissue, resulting in fibrosis and fixation to the surrounding anal mucosa [5, 8]. The most important disadvantages are the feeling of urgency, pain and soiling during and after the procedure. A rare but serious complication of RBL is the occurrence of an arterial bleeding, which in some cases needs to be stopped surgically. On the contrary, SCL induces an inflammation process and local sclerosis of the submucosa which initiates fixation of the haemorrhoidal tissue to the anorectal wall [6, 8]. The haemorrhoid shrinks through the obliteration of the vascular wall. Current literature does not describe major negative side effects, with only a few cases of local infection and haemorrhoidal thrombosis, and very rare complications such as fistula formation and impotence [6, 8]. In the event of haemorrhoidal thrombosis, oral pain medication is usually sufficient; surgical incision of the clot is scarcely required. Another agent that is used in the treatment of HD is phenol. A study showed that it can be equally effective as Aethoxysklerol; however, the use of phenol led to more adverse reactions such as pain, necrosis and ulceration [9].

Furthermore, a recent study has shown a lack of uniformity in the definitions for outcomes, when considering a successful treatment of HD [10]. This results in heterogeneity, limited transparency and hampers the ability to adequately compare results. The most common outcomes in HD studies are pain, blood loss, prolapse and incontinence. Results from a systematic review found no significant difference in the effectiveness of RBL and SCL in treating HD in terms of blood loss, recurrences and complications (73–84% vs 69–88%, 10–18% vs 1.5–29%, and 8–80% vs 34–49%, respectively) [6, 8]. In the present time, similar effectiveness in terms of subjective outcomes (PROMs) and patient experience outcomes, such as satisfaction rates and the subjective experience of a treatment, are important and distinctive measures. These experience measures are scarcely reported in current literature.

In May 2019, the first Core Outcome Set (COS) for HD was developed by the European Society of Coloproctology (ESCP) [11]. The primary outcomes are objective symptoms, complications and recurrences. The patient experience from each treatment will be monitored as a secondary outcome. Symptoms should be scored according to standardized PROMs (blood loss, itchiness, soiling, pain and prolapse). Complications such as incontinence, abscess, anal stenosis and fistula formation need to be reported, together with the recurrence of symptoms (subjective recurrence of disease).

### Objectives

Until now, there are no randomised controlled trials studying the difference in effectivity between RBL and SCL for the treatment of HD. The objective of the THROS trial is primarily to investigate the effectivity of RBL and SCL for patients with grade 1–2 HD in terms of symptom reduction, recurrence and complication rate. Secondary outcomes are patient experience, number of treatments, work leave, crossover rate and subjective symptoms related to recurrence.

## Methods and analysis

### Trial design

The THROS trial is a non-inferiority, multicentre, randomised controlled trial with two parallel groups: RBL vs. SCL. The allocation ratio is 1:1. Crossover is possible for patients who experience no improvement of symptoms after at least two treatments within one treatment arm. It is expected that when two treatments have not reduced symptoms and a third treatment is necessary, both patients and surgeons are inclined to try a new treatment option. So crossover rate is also indicative for treatment success. Also, when patients have a strong preference for one of the two treatment arms and refuse randomisation, then registration in the preference arm is allowed. All patients’ symptoms could not be effectively managed by conservative treatment, which included dietary changes, laxatives, and lifestyle improvements. Conservative management was the first step of therapy in all cases. After eligibility has been established and patients have given their written informed consent, allocation or registration to one of the two treatment arms is possible. Data will be analysed on both ‘intention to treat’ and ‘per protocol’ basis.

The trial was registered at the Dutch Trial Register (NL8377). The protocol was drafted in accordance with the Standard Protocol Items: Recommendations for Interventional Trials statements (SPIRIT) [12].

### Eligibility criteria

#### Inclusion criteria

- Grade 1 (symptomatic with rectal blood loss) or grade 2 haemorrhoids (Golligher classification)
- Aged 18 years old or older and legally competent

#### Exclusion criteria

- Grade 3 and 4 haemorrhoids (Golligher classification)
- Patients that have undergone treatment for HD within the last 12 months, regardless of the type of treatment.

### Interventions

Eligible patients are randomised via Castor^EDC^ to either the SCL or RBL arm. Patients who deny randomisation are eligible to the preference arm of their choice. Patients who are randomised to receive SCL are treated with 4 cc Aethoxysklerol 3% (polidocanol) in the haemorrhoidal tissue via a small 18 mm proctoscope (Sapimed). Patients who are randomised to receive RBL are treated via the same small proctoscope with 3 rubber bands which are placed at the base of the haemorrhoidal tissue (Barron ligation). When after approximately 6–8 weeks a second treatment is required this should be the same treatment as the first (SCL or RBL), after which, in case of persistence after at least two treatments and again 6–8 weeks in between treatments, a crossover is permitted. There are no restrictions regarding concomitant care during the trial.

### Outcomes

#### Primary outcomes

The primary outcome measures are treatment efficacy measured through PROMs according to the ESCP core outcome set of symptom reduction, subjective recurrences and complications (Table 1). The follow-up period for this outcome set is 6 months, and data will be collected at 4 different time points during this period (baseline, 1 week, 6 weeks and 6 months after the first procedure).

**Table 1:** Primary and secondary outcome measures

	**Tools of measurement**
**Primary outcomes**	
*Patient-related outcomes*	PROM (minimal score 5, maximum score 25): The lower the score the better the result
- Blood loss	Likert scale – 1 (not at all) to 5 (very much)
- Pain	Likert scale – 1 (not at all) to 5 (very much)
- Prolapse	Likert scale – 1 (not at all) to 5 (very much)
- Itching	Likert scale – 1 (not at all) to 5 (very much)
- Soiling	Likert scale – 1 (not at all) to 5 (very much)
	HISS (minimal score 0, maximum score 20)
- Impact on daily activities	Scale – 0 (no impact at all) to 10 (highly impacted on daily activities)
- Satisfaction with treatment	Scale – 0 (not at all satisfied) to 10 (very satisfied)
*Complications*	
Incontinence	Wexner Fecal Incontinence Score
Abscess	Physical examination^a^
Fistulation	Physical examination^a^
Urine retention	Bladder scan
Anal stenosis	Physical examination^a^
Anal fissure	Physical examination
Arterial bleeding	Physical examination
Thrombosed haemorrhoid	Physical examination
*Recurrence*	Subjective return of initial symptoms
**Secondary outcomes**	
Patient experience	PREM
Absenteeism	Days of sick leave from work
Number of treatments	Numeric
Crossover rate	Numeric

*PROM* patient-reported outcome measure, *PREM* patient-reported experience measure

^a^If inconclusive follow up with an ultrasound or MRI

#### Treatment efficacy

This outcome follows the patient-reported outcome measure-haemorrhoidal impact and satisfaction score (PROM-HISS) questionnaire from the international Delphi procedure of the European Coloproctology group, for which recently the first steps in the validation process were taken [10, 11, 13].

This questionnaire consists of three domains: (1) HD symptoms, (2) impact of HD on daily activities, and (3) satisfaction with treatment [13]. The first domain contains five HD symptoms of which each is scored on a Likert scale ranging from 1 (“not at all”) till 5 (“very much”), resulting in a maximum possible score of 25. During follow-up, any reduction in score on this numeric scale is classified as ‘improvement’. This will then be translated into a binary outcome: “yes” or “no” improvement of symptoms. Both the second and third domains only contain one item, the impact of HD on daily activities and patients’ satisfaction with treatment, respectively. Both are scored on a 10-point numeric rating scale. In respect to the impact of symptoms on daily activities, 0 correlates with “no impact at all” and 10 with “highly impacted on daily activities”. On the contrary, for patient satisfaction with treatment, this ranges between 0 “not at all satisfied” and 10 “very satisfied”. Data from the PROM-HISS questionnaire is collected 1 week after the procedure, so the recall period comprises “the past week” [13].

Complications (i.e. incontinence, abscess, fistula, fissure, urine retention, anal stenosis, arterial bleeding or thrombosed haemorrhoid) are scored at the 6–8 week clinical follow-up appointment at the outpatient clinic. Subjective return of initial symptoms (recurrence) is recorded during the 6-month follow-up period.

#### Secondary outcomes

Secondary outcome measures consist of patient experience outcomes (PREMs), number of treatments, crossover and number of days on work leave.

Patient experience outcomes are recorded on the PREM questionnaire (Appendix). It contains a series of questions regarding the patients’ experience of the treatment, which is partly scored on a Likert scale ranging from 1 (“not at all”) till 5 (“very much”). Two additional binary (yes/no) questions are added regarding whether patients would undergo the same treatment and whether they would recommend the treatment to acquaintances suffering from similar HD symptoms. The administration of this questionnaire takes place 1 week after treatment. The total number of treatments, the crossover of treatment arms and days of work leave are recorded after a total of 6 months of follow-up. All data entries are stored in Castor^EDC^.

### Participant timeline

Treatment is provided according to the following timeline (Table 2):

- T = day 0 → outpatient clinic treatment with either SCL or RBL after providing written informed consent to the treating physician.
- T = day 7 → telephone appointment with a blinded researcher: the PROM and PREM questionnaires are completed by the researcher.
- T = 6 → weeks outpatient clinic appointment with a physician. The PROM and Wexner incontinence scale are completed together with a physical examination. If needed, the same treatment (SCL or RBL) is repeated.
- T = 6 → months telephone appointment with a blinded researcher: PROM questionnaire completed and (subjective) symptoms related to recurrence are assessed.

**Table 2:** Participant timeline

	**Study period**			
	**Enrolment/allocation**	**Post-allocation**		
**Time point**	Baseline (at allocation or by telephone)	1 week (telephone)	6 weeks (outpatient clinic)	6 months (telephone)
**Eligibility screen**	X			
**Informed consent**	X			
**Allocation (RBL/SCL)**	X		X	
**Primary outcomes**				
HD symptoms (PROM)	X	X	X	X
Abscess			X	
Urine retention			X	
Anal stenosis			X	
Incontinence			X	
Fistula			X	
Recurrence			X	X
**Secondary outcomes**				
Satisfaction/experience (PREM)		X		
Absenteeism		X	(X)	
Number of treatments			X	X
Crossover rate				X

### Sample size calculation

The sample size is based on a success percentage of 70% in both treatment arms, in terms of symptom reduction. Based on the available literature from Cocorullo et al. [6], we will perform a non-inferiority analysis with a two-sided significance (alpha 2.5%), 80% power and a 10% non-inferiority limit. Consequently, a total of 330 randomised patients are required per arm, so 660 randomised patients in total.

- Significance level (a) = 2.5%
- Power (1-beta) = 80%
- Non-inferiority limit = 10%
- Proportionality group A = 70%
- Proportionality group B = 70%
- Sample size required per group = 330
- Total sample size required = 660

### Recruitment and blinding

In the Netherlands patients with HD are mostly referred to the surgeon by their general practitioner. The outpatient clinic nurses or researchers will screen for eligibility and send the patient written information (study information folder (PIF) and plan their appointment at the outpatient clinic. This allows for a minimum of 48 h to reflect on the information. The patient takes the information to the outpatient clinic appointment to discuss with the treating physician. In the case of participation, oral and written consent are gathered.

- The treating physician generates the allocation process or registration to the preference arm, after written informed consent has been provided.
- The randomization sequence will be computer generated with the Castor^EDC^ program (version 24.41) without stratification, with 1:1 allocation to either group, generating a unique record number.
- Both patient and treating physician are not blinded for the treatment arm.
- In concept, the researcher who completes the telephone interviews at 7 days and 6 months is blinded to the given therapy and is instructed not to actively ask patients which treatment they received. However, the data is added in the same database system, so complete blinding is not guaranteed. The physician at T = 0 and T = 6 weeks is not blinded as it is otherwise impossible to complete the treatment. Also, at 6 weeks the treating physician should repeat the initial treatment when necessary, following protocol.

### Data collection

Each participating centre’s personnel involved in treating patients with HD are trained in providing eligible patients with both oral and written information about the study. All the medical baseline and procedure data are collected at the individual hospitals. The data is stored in standardized case record forms (CRFs) within Castor.

The surgeons involved in treating patients with HD are responsible in collecting the data from the initial visit/treatment and the 6-week outpatient visit where complications are scored. Research personnel of each participating centre is responsible for their own data collection during the 6-month follow-up period.

## Statistical analysis

Both intention-to-treat and per-protocol analyses shall be carried out. Crossover is not desirable, but possible if deemed necessary by the treating physician. Crossover will be considered as a secondary outcome measure. Analyses will be carried out using SPSS version 26.0. A *p*-value of 0.05 or less is considered as statistically significant.

For the primary outcome, any reduction in a PROM/PREM score per symptom is scored as ‘improvement’ when considering the results as binary success or not. Analysis of primary outcomes will be tested with chi-square test or Fisher’s exact test. Considering there are four time points for the outcomes, these time points will be taken into account during the analysis. Descriptive methods will be used to check the quality of the data, homogeneity of the two treatment groups and primary and secondary endpoints. Significant confounders will be identified using multivariate analysis. Categorical variables will be presented as percentages and continuous variables will be presented as means with their confidence intervals. To examine gender differences, an additional analysis will be carried out. An interim analysis will take place after the first 300 patients have been randomised and 6-month follow-up is completed. Both the number of patients who experienced complications and the number of patients with recurrent symptoms after 6 months will be evaluated between the two groups. If there are significant differences, then the entire study group will discuss these findings and discuss whether the study can be continued.

### Data monitoring

The THROS trial is monitored internally by the Scientific Office of the Flevoziekenhuis in cooperation with the hospital quality and safety department. All participating centres also ensure monitoring standards. Any serious complications must be reported as soon as possible to the coordinating researchers who will report this to the chairman of the Scientific Office. No external data monitoring committee has been established because the interventions included in this trial are low-risk interventions and have been used in the participating centres for a significant amount of time (i.e. > 10 years). The principal investigator will update the Medical Ethics Committee on the status of the investigation once a year. An estimation of the degree to which the study objectives are reached, the reporting of adverse events, and other reports that may be relevant for the assessment of the investigation's progress are all included in the yearly progress report.

## Ethics and dissemination

### Ethics approval

This trial will be carried out according to the principles of the Declaration of Helsinki (Fortaleza October 2013) and in accordance with the WMO and other European guidelines, regulations and acts such as the GDPR (General Data Protection Regulation) and Good Clinical Practice (GCP). The medical ethics committee approved the protocol of this study on September 24, 2020 (nr. 2020_053). Important modifications from amendments will be communicated to participating centres.

### Consent procedure

The allocation process and consent procedure are described above. Moreover, participation is voluntary and patients are free to discontinue participation and withdraw from the study at any time without giving reason. On the consent form, participants will be asked if they agree to the use of their data should they choose to withdraw from the trial. Participants will also be asked for permission for the research team to share relevant data with people from the centres taking part in the research or from regulatory authorities, where relevant. On the consent form, we also request participants' consent to contact them for ancillary studies.

### Risk of harms

No additional harm or compensation is predicted for trial participation since both treatment choices are part of approved clinical practice for HD. However, of course, the principal investigator and his team will report all serious adverse events (SAEs) to the accredited medical ethical committee. In addition, once a year throughout the trial, the study group will submit a safety report to the accredited MEC.

### Confidentiality/access to data

After allocation to one of the treatment arms within Castor^EDC^ an unique record number is regenerated. Original data and the decoding key will be stored at the patients’ clinic and must be handled with care. The data will be saved for 15 years and only members from the research group will have access to the study data. The final trial data for this protocol can be provided upon reasonable request.

### Methods of dissemination of results

Results from this study will be communicated through peer-reviewed medical journals and data can be made available upon reasonable request.

## Discussion

The THROS study is the first large multicentre randomised trial to study the difference in effectivity between RBL and SCL for the treatment of grade 1–2 HD. Primary and secondary outcomes consist of both objective and subjective patient-reported outcomes. In this non-inferiority trial, we hypothesize that SCL is non-inferior to RBL with regard to effectiveness, patient experience, complications and recurrences.

As HD is one of the most common conditions in the general population, with an annual incidence of 5%, it represents a significant epidemiologic and economic burden. Most patients experience blood loss and for some patients, this blood loss can cause symptomatic anaemia. Patients with grade 1–2 haemorrhoids may also experience other symptoms like itchiness, pain or soiling and, because of additional stigma and shame, it represents a high burden that affects the quality of life.

However, patients’ experience of HD symptoms and its burden on everyday life is in contrast with the surgeon’s experience in which it is perceived as a ‘simple’ diagnosis with a relatively ‘simple’ solution/treatment. Therefore, surgeons and proctologists are driven to treat their patients with less invasive, but most effective method, in which the patients’ experience of the treatment also plays an important role. This is especially so considering that for the majority of cases, a singular treatment is insufficient to fully treat the HD.

In case of more severe (grade 3–4) or recurrent HD, there are several multicentre randomised controlled trials studying the effect of different treatment options. Both the HollAND and Napoleon trial, which were forced to stop prematurely due to inclusion difficulties, are currently in the follow-up phase. Both studies compare more invasive techniques (haemorrhoidectomy or sutured mucopexy) with RBL [14, 15]. However, symptomatic grade 1–2 HD are far more common in the general population and so an answer as to what the best and most appropriate minimal invasive treatment option is, is needed.

For the treatment of grade 1–2 HD, there are several options described in the literature and some of them are relatively new [16–19]. The THROS trial focuses on two less invasive treatment methods and it will provide us with information as to which commonly used treatment method (RBL or SCL) of grade 1–2 HD is the most effective, gives fewer complications and is experienced by the patient as the best option. Considering the available literature on SCL and RBL, we expect the efficacy and complication rates to be equal. However, in most cases of SCL or RBL therapy, repeated procedures are necessary to achieve acceptable symptom reduction, and it is for that reason that it is very important to investigate the patients’ experience of treatment. In current literature, there is limited available evidence regarding patient experience, except for pain scores [6]. Therefore, it is of even greater importance to compare both treatments in other domains, with the use of the recently developed PROM-HISS questionnaire [11, 13].

### Supplementary Information

**Additional file 1: ** Patient Information Sheet.

## Appendix

Patient-reported experience measure (PREM):

1. How much pain did you experience DURING the treatment on a scale of 0 (none) to 4 (very much)?: 0–4
2. How much pain did you experience AFTER the treatment?: 0–4
3. How much feeling of URGE did you experience AFTER the treatment?: 0–4
4. How satisfied were with the treatment on a scale of 1 (very satisfied) to 5 (very unsatisfied)?: 1-5
5. Would you, if necessary, undergo this treatment again?: yes/no
6. Would you recommend this treatment to acquaintances with the same symptoms?: yes/no
