# Supplementary material for: Treatment of haemorrhoids: rubber band ligation or sclerotherapy (THROS)? Study protocol for a multicentre, non-inferiority, randomised controlled trial
Source: Trials. 2023 Jun 3;24:374. doi: 10.1186/s13063-023-07400-2 (PMC10239572; doi:10.1186/s13063-023-07400-2)
Supplement: Supplementary file 1 — Additional file 1: Patient Information Sheet. [file 13063_2023_7400_MOESM1_ESM.pdf]

Proefpersoneninformatie voor deelname aan medisch-wetenschappelijk onderzoek

__T__reatment of __H__emorrhoids: __R__ubberband ligation __O__r __S__clerotherapy (THROS-trial)

Rubberbandligatie versus sclerotherapie voor graad 1-2 hemorroïden: symptoombestrijding en patiëntbeleving

Inleiding

Geachte heer/mevrouw,

Wij vragen u om mee te doen aan een medisch-wetenschappelijk onderzoek. Meedoen is vrijwillig. Om mee te doen is wel uw schriftelijke toestemming nodig. U ontvangt deze brief omdat u een behandeling voor aambeien zult ondergaan. Voordat u beslist of u wilt meedoen aan dit onderzoek, krijgt u uitleg over wat het onderzoek inhoudt. Lees deze informatie rustig door en vraag uw arts uitleg als u vragen heeft. U kunt er ook over praten met uw partner, vrienden of familie.

1. Algemene informatie

Dit onderzoek wordt gedaan door het Flevoziekenhuis ziekenhuis in opdracht van dr. R. Schouten en dr. S. Smeets, gastro-intestinale en proctologische chirurgen van het Flevoziekenhuis te Almere. Algemene informatie over het meedoen aan medisch onderzoek vindt u op de internetsite van de rijksoverheid (www.rijksoverheid.nl/mensenonderzoek).

2. Doel van het onderzoek

Het doel van dit onderzoek is om twee behandelingen van aambeien te vergelijken; rubberband-ligatie versus sclerotherapie. Hierbij kijken we naar patiënten tevredenheid van de behandeling, het opnieuw optreden van de kwaal, complicaties, pijn na de behandeling en de tijd tot het hervatten van de dagelijkse bezigheden. Deze uitkomsten worden al standaard met u besproken tijdens de

reguliere poliklinische afspraken bij de chirurg. Daarnaast vragen we u ook om vragen te beantwoorden op diverse momenten voor en na de behandeling.

3. Achtergrond van het onderzoek

Er bestaan verschillende chirurgische technieken voor de behandeling van aambeien, zowel het ombinden van de aambei met rubberbandjes als het uitlokken van een litteken reactie door ze in te spuiten met de vloeistof aethoxysklerol. Onderzoek laat tot nu toe vergelijkbare resultaten zien van de twee behandelmethodes. Wij zouden graag het antwoord vinden op de vraag welke behandeling voor u als patiënt het minst onprettig is.

4. Wat meedoen inhoudt

Middels deze brief vragen wij uw toestemming om de resultaten van de aambei behandeling, gedurende uw behandeltraject, te verzamelen. U zal 1 van de 2 behandelingen ondergaan en het lot zal bepalen welke behandeling dat wordt. Als u wel wilt meedoen aan het onderzoek maar niet geloot wilt worden omdat u een duidelijke voorkeur heeft voor een type behandeling dan is dat mogelijk. Dan komt u in de ‘voorkeursarm’ (preference arm)’ van het onderzoek. U krijgt dan de behandeling die u wenst maar kan wel meedoen aan het verdere onderzoek en de vragenlijsten. Hierbij willen wij kijken naar gegevens relevant aan uw aandoening zoals complicaties, her-operaties, pijn na de operatie en tijd tot het hervatten van de dagelijkse activiteiten. Daarnaast vragen wij u om op 4 momenten vragen te beantwoorden over uw kwaliteit van leven en uw fysieke inspanning voor en na de ingreep. De eerste vragen worden door uw arts gesteld vóórdat u de behandeling ondergaat. Zeven dagen na de ingreep ontvangt u een telefoontje waarop wederom een rijtje vragen worden gesteld. Bij uw poliklinische bezoek na 6 weken zal uw arts deze weer vragen en na 6 maanden ontvangt u het laatste telefoontje. De telefoongesprekken duren ongeveer 5 tot 10 minuten per keer.

Als u meedoet aan dit onderzoek heeft dit geen positief of negatief resultaat op uw ziekte. Maar u draagt wel bij aan meer kennis over de behandeling van aambeien. Voor dit onderzoek wordt 7 dagen na de behandeling telefonisch contact met u gezocht. Normaal gesproken zou dat niet gebeuren dus dit betreft een extra

controle. Zes weken na de behandeling wordt u op de polikliniek gezien (standaard afspraak, dat zou ook gebeuren als u niet meedoet aan het onderzoek) en na 6 maanden wordt u wederom gebeld (extra in het kader van het onderzoek; als u geen klachten heeft hoeft u niet naar het ziekenhuis te komen). Indien er gegevens missen die noodzakelijk zijn voor de resultaten van deze studie wordt er telefonisch contact opgenomen met u.

5. Wat wordt er van u verwacht

Wij vragen u om de vragenlijsten in te vullen. Er zijn geen extra poliafspraken of onderzoeken nodig. Er wordt wel extra telefonisch contact met u gezocht 7 dagen en 6 maanden na de behandeling.

Om het onderzoek goed te laten verlopen, is het belangrijk dat u zich aan de volgende afspraken houdt.

De afspraken zijn dat u

- Niet ook nog aan een ander medisch-wetenschappelijk onderzoek meedoet.
- Afspraken voor bezoeken nakomt.

Het is belangrijk dat u contact opneemt met de onderzoeker:

- Als u in een ziekenhuis wordt opgenomen of behandeld.
- Als u plotseling gezondheidsklachten krijgt.
- Als u niet meer wilt meedoen aan het onderzoek.
- Als uw contactgegevens wijzigen.

6. Mogelijke bijwerkingen/complicaties en andere/ nadelige effecten/ ongemakken

De te onderzoeken ingrepen kunnen bijwerkingen geven.

- Bloedverlies
    - Dit is bijna nooit ernstig en gaat meestal vanzelf over. Indien u bloed verliest en twijfelt vragen wij u telefonisch contact op te nemen met ons.
- Pijn
    - U kunt pijn verwachten bij deze ingreep; hiervoor bestaan goede pijnstillers. Deze adviseren wij u te gebruiken.
- Incontinentie

        - Normaal gesproken zou incontinentie niet op moeten treden na een operatie. Dit risico bestaat wel als de sluitspier al minder goed werkt door een bevalling, eerdere operatie of bestraling. Een gespecialiseerd arts zal dit vooraf altijd controleren.
    - Recidief (herhaling van de klachten)
        - De kans de aambeien na de operatie terugkomen is kleiner als de stoelgang normaal is en u niet hoeft te persen naast de andere adviezen welke bij u meegegeven.

Indien u een van de bovenstaande klachten meemaakt neem gerust contact op met de polikliniek chirurgie.

7. Mogelijke voor- en nadelen

Het is belangrijk dat u de mogelijke voor- en nadelen goed afweegt voordat u besluit mee te doen.
U heeft zelf geen voordeel van meedoen aan dit onderzoek. Uw deelname kan wel bijdragen aan meer kennis over de behandeling.

Nadelen van meedoen aan het onderzoek kunnen zijn:

- Mogelijke bijwerkingen/complicaties van de twee behandelopties
- U kunt niet zelf een keuze maken of voorkeur opgeven voor behandeling

Deelname aan het onderzoek betekent ook:

- Dat u extra tijd kwijt bent
- Het invullen van vragenlijsten
- Dat u afspraken heeft waaraan u zich moet houden;

Al deze zaken zijn hiervoor onder punt 4, 5 en 6 beschreven.

8. Als u niet wilt meedoen of wilt stoppen met het onderzoek

U beslist zelf of u meedoet aan het onderzoek. Deelname is vrijwillig. Dit onderzoek heeft geen invloed op uw huidig behandeltraject. Indien u niet meedoet aan het onderzoek zal u de behandeling van uw voorkeur krijgen.

Als u wel meedoet, kunt u zich altijd bedenken en toch stoppen, ook tijdens het onderzoek. U hoeft niet te zeggen waarom u stopt. Wel moet u dit direct melden aan uw arts. De gegevens die tot dat moment zijn verzameld, worden gebruikt voor het onderzoek.

Als er nieuwe informatie over het onderzoek is die belangrijk voor u is, laat uw arts dit aan u weten. U wordt dan gevraagd of u blijft meedoen.

9. Einde van het onderzoek

Uw deelname aan het onderzoek stopt als u alle vragenlijsten heeft ingevuld en als alle relevante informatie wat betreft uw aandoening waarvoor u de behandeling hebt gekregen uit uw elektronisch patiëntendossier is gehaald. Het hele onderzoek is afgelopen als de vragenlijsten van alle deelnemers ingevuld zijn en alle relevante informatie verzameld is.

10. Gebruik en bewaren van uw gegevens

Het onderzoek is online geregistreerd op de site van het Nederlandse trial register, (https://www.trialregister.nl/trial/8377). Op deze site zijn alle Nederlandse onderzoeken inzichtelijk en voor iedereen toegankelijk.
Voor dit onderzoek worden uw persoonsgegevens gebruikt en bewaard. Het verzamelen, gebruiken en bewaren van uw gegevens is nodig om de vragen die in dit onderzoek worden gesteld te kunnen beantwoorden en de resultaten te kunnen publiceren. Wij vragen voor het gebruik van uw gegevens uw toestemming.

Vertrouwelijkheid van uw gegevens
Om uw privacy te beschermen krijgen uw gegevens een code. Uw naam en andere gegevens die u direct kunnen identificeren worden daarbij weggelaten. Alleen met de sleutel van de code zijn gegevens tot u te herleiden. De sleutel van de code blijft veilig opgeborgen in de Proctos Kliniek. Ook in rapporten en publicaties over het onderzoek zijn de gegevens niet tot u te herleiden.

Toegang tot uw gegevens voor controle
Sommige personen kunnen op de onderzoekslocatie toegang krijgen tot al uw gegevens. Ook tot de gegevens zonder code. Dit is nodig om te kunnen controleren

of het onderzoek goed en betrouwbaar is uitgevoerd. Personen die ter controle inzage krijgen in uw gegevens zijn: nationale en internationale toezichthoudende autoriteiten, bijvoorbeeld, de Inspectie Gezondheidszorg en Jeugd. Zij houden uw gegevens geheim. Wij vragen u voor deze inzage toestemming te geven.

Bewaartermijn gegevens
Uw gegevens moeten 15 jaar worden bewaard op de onderzoekslocatie.

Uw gegevens kunnen na afloop van dit onderzoek ook nog van belang zijn voor ander wetenschappelijk onderzoek op het gebied van de behandeling van aambeien. Daarvoor zullen uw gegevens 15 jaar worden bewaard. U kunt op het toestemmingsformulier aangeven of u hier wel of niet mee instemt. Indien u hier niet mee instemt, kunt u gewoon deelnemen aan het huidige onderzoek.

Intrekken toestemming
U kunt uw toestemming voor gebruik van uw persoonsgegevens altijd weer intrekken. Dit geldt voor dit onderzoek en ook voor het bewaren en het gebruik voor het toekomstige onderzoek. De onderzoeksgegevens die zijn verzameld tot het moment dat u uw toestemming intrekt worden nog wel gebruikt in het onderzoek.

Meer informatie over uw rechten bij verwerking van gegevens
Voor algemene informatie over uw rechten bij verwerking van uw persoonsgegevens kunt u de website van de Autoriteit Persoonsgegevens raadplegen.

Bij vragen over uw rechten kunt u contact opnemen met de verantwoordelijke voor de verwerking van uw persoonsgegevens. Voor dit onderzoek is dat: dr. Schouten.

Bij vragen of klachten over de verwerking van uw persoonsgegevens raden we u aan eerst contact op te nemen met het onderzoeksteam. U kunt ook contact opnemen met de Functionaris voor de Gegevensbescherming van de instelling kunt u vinden in **bijlage A** of de Autoriteit Persoonsgegevens.

11. Verzekering voor proefpersonen

Als u deelneemt aan het onderzoek loopt u dezelfde risico's als bij de gebruikelijke behandeling van uw aandoening. De onderzoeker hoeft daarom van de toetsende commissie geen extra verzekering af te sluiten.

12. Informeren huisarts

Wij zullen uw huisarts niet informeren dat u met ons onderzoek meedoet.

13. Geen vergoeding voor meedoen

Er zijn geen extra kosten en of vergoedingen verbonden aan het meedoen van het onderzoek.

14. Heeft u vragen?

Bij vragen kunt u contact opnemen met het onderzoeksteam.
Indien u klachten heeft over het onderzoek, kunt u dit bespreken met de onderzoeker of uw behandelend arts. Wilt u dit liever niet, dan kunt u zich wenden tot de klachtenbemiddelaar van de Proctos Kliniek. Alle gegevens vindt u in **bijlage A**: Contactgegevens.

15. Ondertekening toestemmingsformulier

Wanneer u voldoende bedenktijd heeft gehad, wordt u gevraagd te beslissen over deelname aan dit onderzoek. Indien u toestemming geeft, zullen wij u vragen deze op de bijbehorende toestemmingsverklaring schriftelijk te bevestigen. Deze kunt u vinden in **bijlage B**. Door uw schriftelijke toestemming geeft u aan dat u de informatie heeft begrepen en instemt met deelname aan het onderzoek.

Het handtekeningenblad wordt door de onderzoeker bewaard. Zowel uzelf als de onderzoeker ontvangen een getekende versie van deze toestemmingsverklaring(en).

Dank voor uw aandacht.

Bijlage A: contactgegevens

Per deelnemend centrum verschillend.

Bijlage B: toestemmingsformulier proefpersoon

Rubberbandligatie versus sclerotherapie voor graad 1-2 hemorroïden: symptoombestrijding en patiëntbeleving (THROS-trial)

- Ik heb de informatiebrief gelezen. Ook kon ik vragen stellen. Mijn vragen zijn voldoende beantwoord. Ik had genoeg tijd om te beslissen of ik meedoe.
- Ik weet dat meedoen vrijwillig is. Ook weet ik dat ik op ieder moment kan beslissen om toch niet mee te doen of te stoppen met het onderzoek. Daarvoor hoef ik geen reden te geven.
- Ik weet dat voor de controle van het onderzoek sommige mensen toegang tot al mijn gegevens kunnen krijgen. Die mensen staan vermeld in deze informatiebrief. Ik geef toestemming voor die inzage door deze personen.

- Ik geef □ wel

  □ geen

  toestemming om te loten ("randomiseren") tussen de twee behandelingen

- Ik geef □ **wel**

  □ **geen**

  toestemming om mijn persoonsgegevens langer te bewaren en te gebruik

  voor toekomstig onderzoek op het gebied van de behandeling bij aambeien

- Ik geef □ **wel**

  □ **geen**

  toestemming om mij na dit onderzoek opnieuw te benaderen voor een vervolgonderzoek

- Ik wil meedoen aan dit onderzoek.

Naam proefpersoon:

Geboortedatum:

Handtekening: Datum: __ / __ / __

Ik verklaar dat ik deze proefpersoon volledig heb geïnformeerd over het genoemde onderzoek.

Als er tijdens het onderzoek informatie bekend wordt die de toestemming van de proefpersoon zou kunnen beïnvloeden, dan breng ik hem/haar daarvan tijdig op de hoogte.

Naam onderzoeker (of diens vertegenwoordiger):
Handtekening: Datum: __ / __ / __

De proefpersoon krijgt een volledige informatiebrief mee, samen met een getekende versie van het toestemmingsformulier.
